# Pathophysiology of Swallowing Dysfunction in Parkinson Disease and Lack of Dopaminergic Impact on the Swallow Function and on the Effect of Thickening Agents

**DOI:** 10.3390/brainsci10090609

**Published:** 2020-09-04

**Authors:** Weslania Viviane Nascimento, Viridiana Arreola, Pilar Sanz, Ediz Necati, Mireia Bolivar-Prados, Emilia Michou, Omar Ortega, Pere Clavé

**Affiliations:** 1 Medical School of Ribeirao Preto, University of São Paulo, São Paulo 14049-900, Brazil; wdo@csdm.cat; 2 Gastrointestinal Physiology Laboratory, CIBERehd CSdM-UAB, Hospital de Mataró, 08404 Mataró, Spain; oarreola@csdm.cat (V.A.); mbolivar@csdm.cat (M.B.-P.); oortega@csdm.cat (O.O.); 3 Centro de Investigación Biomédica en Red, Enfermedades Hepato-Digestivas (CIBERehd) Instituto de Salud Carlos III, 28029 Madrid, Spain; 4 Neurology Department, Hospital de Mataró, Universitat Autònoma de Barcelona, 08404 Mataró, Spain; psanzcart@gmail.com; 5 Department of Physiotherapy and Rehabilitation, Faculty of Health Sciences, Near East University, Nicosia 99138, Cyprus; ediz.necati@neu.edu.tr; 6 Department of Speech Language Pathology: Communication Disorders and Dysphagia, University of Patras, 26334 Patras, Greece; emiliamichou@upatras.gr

**Keywords:** oropharyngeal dysphagia, Parkinson’s disease, oropharyngeal swallow response, thickening agents, dopamine, shear viscosity, OFF/ON states

## Abstract

(1) Background: The effect of dopaminergic treatment on swallowing response in patients with Parkinson’s disease (PD) suffering oropharyngeal dysphagia (OD) is not understood. Aim: To characterize OD pathophysiology in PD and to assess whether dopaminergic states affect swallow function and the effect of thickeners. (2) Methods: Fifty patients with PD (40 evaluated in OFF/ON states) and 12 healthy volunteers (HVs) were evaluated with videofluoroscopy (VFS) to assess the swallowing biomechanics and kinematics of the swallowing response at three different shear-viscosities (<50, 120, and 4000 mPa·s); (3) Results: Patients presented a mean age of 70.46 ± 10.03 years. Disease evolution was 5.09 ± 3.86 year and Hoehn-Yahr stage was 2.32 ± 0.81. For HVs, mean age was 40.20 ± 2.50 year. Penetrations were present in 37.50% of PD patients and were associated with delayed laryngeal vestibule closure (LVC = 293.33 ± 90.07 ms). In contrast, HVs presented a LVC = 164.00 ± 39.78 ms (*p* < 0.05). An LVC ≥ 260 ms cutoff predicted unsafe swallow (sensitivity ≥ 0.83, specificity ≥ 0.57, AUC = 0.80) in PD. Increasing bolus viscosity improved deglutition safety but increased oropharyngeal residue. There were no differences in swallowing between the OFF/ON states. (4) Conclusions: In initial PD stages, oropharyngeal swallow response is severely delayed, while mildly impaired swallow safety improves with increasing bolus viscosity, which increases residue. Dopaminergic treatment does not affect swallowing or the therapeutic effect of thickeners.

## 1. Introduction

Patients with Parkinson’s disease (PD) frequently have an impaired swallowing function [1,2,3,4]. Oropharyngeal dysphagia (OD) prevalence among patients with PD, which has been reported to vary between 11% and 82%, is related to disease stage, disease duration, and diagnostic methods [2,5]. Impaired swallow safety in PD is linked with lagged airway protection due to the delay in the laryngeal vestibule closure (LVC) time caused by both central neurological motor impairment and sensory loss in the pharynx and larynx [6]. Impaired swallow efficacy, associated with weak tongue squeeze, weak bolus propulsion, incomplete upper esophageal sphincter (UES) relaxation, and reduced UES compliance is encountered in up to 21% patients with PD and is caused by bradykinesia, shaking, weakness, and extrapyramidal rigidity [7,8]. The neurophysiological basis for those severe dysfunctions has been recently revised by our group [1].

OD affects health and quality of life and interferes with the oral intake of medication in patients with PD [9,10], even in early stages of the disease. These patients are therefore often at risk of malnutrition, dehydration, aspiration pneumonia, and increased mortality [1,11]. Information on OD pathophysiology, its natural course in PD, and PD impact on swallowing stages is lacking [1]. Doubts also remain regarding the optimal application of swallowing therapy (early vs. late, active vs. compensatory, etc.) and the role played by dopaminergic treatment and fluid thickening. A further issue is that healthcare provider awareness of the importance of OD in PD needs to be raised.

The role of dopaminergic medication for swallowing dysfunction in PD is still controversial, particularly in the early disease stages [1]. Previous studies have reported improved swallowing in response to levodopa administration in patients at advanced disease stages [12,13,14,15,16]. However, no differences have been detected in relation to the prevalence of impaired swallow safety [17,18]. A pilot study has reported decreased swallow efficacy associated with levodopa medication [18]. Cereda et al. (2014) [19] noted that OD is a non-levodopa-responsive motor symptom associated with a high risk of mortality. Michou [20] described that the heterogeneity in the response to levodopa found in the literature might be attributed to the fact whether the neurodegeneration had reached the bulbar area or not, and the different levels of degeneration in the cases with bulbar involvement.

While fluid thickening is a validated therapeutic strategy for patients with OD [21], there is a lack of information regarding the therapeutic effect for patients with PD and OD. Studies have reported various findings, for instance, that pudding-thick fluids compared to liquids increased swallow safety, but also increased transit time and the number of tongue pumps [22]; that a jelly consistency avoided aspiration [23]; and that aspiration avoidance occurred most often with honey-thickened liquids, followed by nectar-thickened liquids and chin-down posture [24], while none of those interventions influenced pneumonia incidence [25]. A more recent study has accurately described the therapeutic shear viscosity range (mPa·s) for patients with PD and OD [26].

Our hypothesis is that the swallowing function in patients with early-stage PD is severely impaired. Given that the relationship between dopaminergic medication and swallow is poorly understood, our aims were as follows: (1) to describe the pathophysiology and main physiological variables of OD in early PD stages; (2) to describe the impact of dopaminergic medication on swallowing; and (3) to describe the effects on swallowing of three shear viscosity levels for thickened fluids.

## 2. Materials and Methods

### 2.1. Study Population

Included were 50 subjects with PD in the early-intermediate disease stages according to the Hoehn-Yahr (1967) criteria [27], recruited through the Neurology Department of the Hospital of Mataró (Catalonia, Spain). Inclusion criteria were adults aged 18 years and older with a confirmed diagnosis of PD according to the Brain Bank of London criteria [28]. Exclusion criteria were patients with severe dementia (Global Deterioration Scale (GDS) > 5) [29], previous stroke, brain damage, or other neurodegenerative diseases, a psychiatric disorder (other than depression) diagnosed before PD, previous surgical treatment for PD, and a life expectancy of less than three months. Patients presenting psychiatric disorder were excluded from the study due to the fact that antipsychotic drugs were dopamine antagonists and can interfere with the PD treatment, and thus with the dopamine effect analysis. In contrast, antidepressants did not act as a dopamine antagonist. Recruited from the community as a control group for comparative purposes were 12 healthy volunteers (HVs) aged 18 years or older. Main inclusion criteria for HVs were: healthy subjects 18–65 years with no swallowing complain, no disease that could deal with swallow impairment and no medication that could influence swallow process; and as exclusion criteria any disease, surgery, or complaint related to swallowing problems. All patients and HVs were informed about the purposes and aims of the study and granted their written informed consent to participate. The study was approved by the Ethics Committee Hospital in May 2014 and registered under the code 17/14.

### 2.2. Drug Analysis

The global dopamine dose was calculated according to Cervantes-Arriaga et al. 2009 [30] based on the different drug compositions and mechanisms of action. The Parkinson drugs that were taken into account and collected for the dopaminergic quantification analysis were: l-Dopa, Dopamine agonists (pramipexole, ropinirole, rotigotina, apomorphine), Mono amino oxidase inhibitors (rasagiline), *N*-methyl-d-aspartate antagonists (amantadine), acetyl choline esterase inhibitors (donepezil, galantamine, rivastigmine), and Duodopa (carbidopa in combination with levodopa; a non-competitive carboxylase inhibitor and a dopamine precursor, respectively). Other drugs collected for the study were corticoids, non-steroidal anti-inflammatory drugs, benzodiazepines, metoclopramide, antidepressants, and B vitamin.

### 2.3. Experimental Design

**Figure 1:**
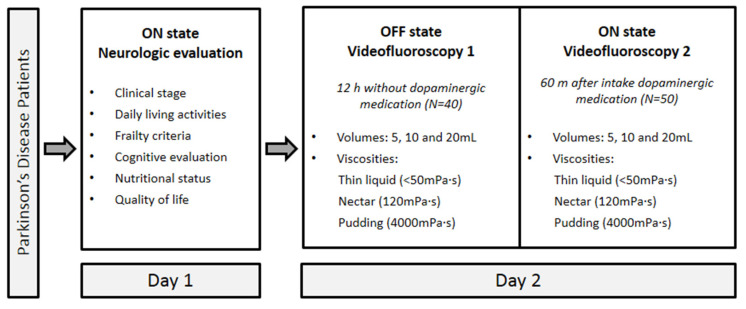
Two-day study design: day 1, clinical and neurological evaluation in ON; day 2, VFS1 in OFF and VFS2 in ON.

This cross-sectional observational comparative two-day study to assess OD pathophysiology in early PD stages included 50 PD patients: 40 patients were assessed in both OFF state (12 h without their dopaminergic medication) and ON state (60 min after dopaminergic medication), in other words, the time required for maximum plasma concentration; 10 patients were assessed in ON status but rejected to stop their daily dopaminergic treatment. The dopaminergic medication was prescribed by the patient’s neurologist at the usual dose. The experimental protocol was as follows (Figure 1): Healthy volunteers (HV, *N* = 12) underwent only one videofluorocopy (VFS) because they were not taking any dopaminergic medication.

#### 2.3.1. Day 1

After signature of the informed consent form, data were collected on sociodemographics (age, sex, education, and family support), alcohol consumption and tobacco use, comorbidities, medication, disease history, and PD symptoms. A comprehensive clinical and neurological evaluation was then conducted by a neurologist, as follows:

(1) PD clinical stage, following Hoehn-Yahr (1967) criteria [27];
(2) Autonomy, according to the Schwab-England Activities of Daily Living (ADL) scale [31];
(3) Non-motor and motor experiences of daily living and motor complications, according to the Movement Disorder Society-Sponsored Revision of the Unified Parkinson’s Disease Rating Scale (MDS-UPDRS) [32];
(4) Frailty status, according to Fried criteria [33];
(5) Cognitive evaluation, according to the Montreal Cognitive Assessment (MoCA) [34];
(6) Nutritional status, according to Mini Nutritional Assessment Short Form (MNA-SF) [35]; and
(7) Health status and quality of life, according to the Parkinson’s Disease Questionnaire-8 (PDQ-8) [36].

#### 2.3.2. Day 2

OFF state. A first videofluoroscopy (VFS1) was performed at 9.00 am, after which patients were asked to take their usual dopaminergic medication. ON state. A second VFS (VFS2) was performed after 60 min (at 10.00 a.m.).

#### 2.3.3. Videofluoroscopy (VFS)

VFS was performed while participants were seated in a lateral position. A Super XT-20 Toshiba Intensifier (Toshiba Medical Systems Europe, Zoetermeer, The Netherlands) recorded 25 frames/second with a Panasonic AG DVX-100B video camera (Matsushita Electric Industrial Co., Osaka, Japan). Images were taken of the oral cavity, pharynx, larynx, and cervical esophagus. Our VFS algorithm was as follows (the full procedure and details have been described elsewhere [37]): Intake started with nectar (120 mPa·s), continued with thin liquids (<50 mPa·s) and pudding (4000 mPa·s) in the same volumetric order of 5 mL, 10 mL, and 20 mL. For each volume and viscosity level, signs of deglutition were identified as:

- Efficacy was evaluated by the presence of oral, vallecular, and pyriform sinus residues, piecemeal deglutition, oral apraxia, pumping, and impaired oral bolus control. Residue was categorized as coating or pooling residue [38].
- Safety included the evaluation of laryngeal vestibule (LV) penetrations and tracheobronchial aspirations, which were assessed for each deglutition and classified according to the Penetration–Aspiration Scale (PAS) [39]. A PAS score ≥2 was considered to describe an unsafe swallow [8].

Swallowing Observer software (Image and Physiology SL, Barcelona, Spain), managed by an expert clinician [40], was used for VFS image digitization, measurements, and analyses. Oropharyngeal swallow response (OSR) was quantitatively measured for swallows of 5 mL of nectar viscosity (120 mPa·s). Timing of opening and closing (indicated by an O or C suffix in the abbreviations that follow) of the glossopalatal junction (GPJ), velopharyngeal junction (VPJ), LV, and upper oesophageal sphincter (UES) was measured, with GPJO given the time value 0. Total OSR duration was calculated as the difference between GPJO and LVO (GPJO–LVO), oropharyngeal reconfiguration from a respiratory to a digestive pathway was calculated as the difference between GPJO and LVC (GPJO–LVC), and the time to closing the UES was calculated as the difference between GJPO and UESO (GJPO–UESO). We also calculated the bolus kinematics (velocity in m/s) according to bolus transit from the GPJ to the UES [40].

#### 2.3.4. Bolus Rheology

Bolus rheology for the VFS study was assessed by a HAAKE Viscotester 550TM viscometer (Thermo Electron, Dieselstraβe 4, D-76227 Karlsruhe, Germany). All boluses were prepared as a 1:1 100 mL solution with mineral water and Gastrografin X-Ray contrast (Bayer Hispania SL, Sant Joan Despí, Spain). A starch-based thickener, Resource ThickenUp (TU) (Nestlé Nutrition, Barcelona, Spain), was added to the solution to prepare nectar and pudding viscosities (3.5 g and 8 g, respectively). Viscosity values for the VFS study were assessed for a shear rate range of 0 s^−1^ to 1000 s^−1^ to ensure the evaluation of the rheological properties of the thickening agent in the entire oropharyngeal deglutition process.

At a shear rate of 50 s^−1^, viscosity values were <50 mPa·s, 117.74 mPa·s ± 25.74, and 4086.97 mPa·s ± 1174.78 (henceforth referred to as 120 mPa·s and 4000 mPa·s, respectively) for the thin liquid, nectar, and pudding viscosities, respectively. The liquid x-ray contrast and water mixture behaved as a Newtonian fluid, viscosity remained constant with increments in the shear rate [41]. Whereas the addition of the starch-based thickening agent to the x-ray contrast solution mixed with water caused non-Newtonian shear-thinning behavior, resulting in a viscosity decrease while the shear rate was increased.

**Figure 2:**
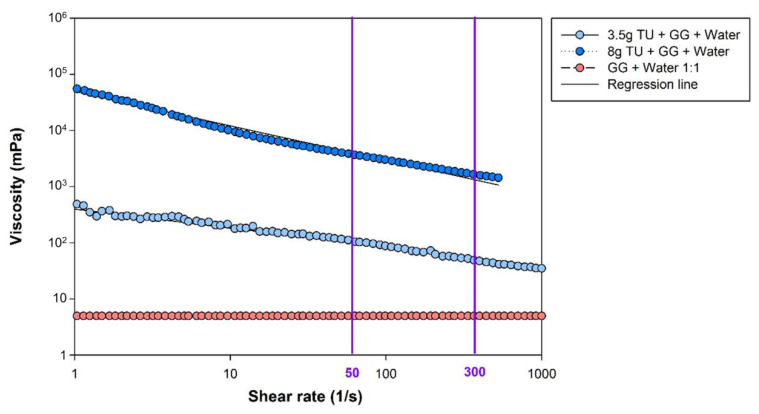
Shear viscosity plots obtained for the shear rate range from 1 s^−1^ to 1000 s^−1^. Purple vertical lines represent the main viscosity points along the shear rate values of 50 s^−1^ (oral phase) and 300 s^−1^ (pharyngeal phase). The orange line reflects the Newtonian behavior of the liquid x-ray contrast mixed with water (1:1 GG and water, <50 mPa·s at 50 s^−1^) and the dark and light blue lines shows the Non-Newtonian shear thinning behavior of 120 mPa· and 4000 mPa·s, respectively. Abbreviations: GG, Gastrografin; TU, ThickenUp.

Regression lines for 120 mPa·s and 4000 mPa·s viscosity in a shear rate range from 0 s^−1^ to 1000 s^−1^ were f (3.5g) = −0.20x + 2.28 (r^2^ (3.5g) = 0.77) and f (8g) = −0.61x + 4.69 (r^2^ (8g) = 0.977), respectively. Viscosity values at the pharyngeal shear rate (300 s^−1^) were extrapolated from the regression lines [42], achieving a value of 57.93 mPa·s and 1474.90 mPa·s for 120 mPa·s and 4000 mPa·s, respectively (Figure 2).

### 2.4. Data Analysis and Statistical Methods

Quantitative data were described as mean ± standard deviation (SD), and comparisons were evaluated using the non-parametric Mann–Whitney U-test. Qualitative or categorical data were described as absolute and relative frequencies and compared using Fisher’s exact test. Swallow safety data were treated as binary (safe/unsafe) and calculated for the total number of patients. Swallow efficacy data were assessed by the presence or absence of various signs such as residues, lip seal impairment, bolus control, etc. [40]. The statistical methods we used to compare the swallow response between HV, PD in ON status, and PD in OFF status were: (a) First, to assess the normality of the results distribution, we used the D’Agostino and Pearson omnibus normality test; and (b) as data did not follow a normal distribution, we used the nonparametric test Kruskal–Wallis with a post-hoc test (Dunns Multiple Comparison test) to compare the swallow response of the three groups. Viscosity levels were compared using the non-parametric McNemar procedure to assess the effect of increases; the null hypothesis for related samples was that multiple responses came from the same population. Receiver-operator characteristic (ROC) curves were plotted to explore the cut-off for LVC time and to distinguish between patients with impaired swallow safety (PAS > 2 or PAS ≥ 2; 120 mPa·s), with diagnostic accuracy reflected as the area under the curve (AUC). The effect of increasing bolus volumes (5 mL to 10 mL to 20 mL) was evaluated using a general linear model (GLM) applied separately for the three viscosities, for the efficacy and safety parameters, and for the ON and OFF states. Statistical significance was set to *p* < 0.05 (non-significance is indicated as ‘ns’). Statistical analyses were performed using GRAPHPAD PRISM 4 (San Diego, CA, USA).

## 3. Results

### 3.1. Demographics and Clinical Inventory Scores

The included 50 patients (24 women) with PD had a mean age of 70.46 ± 10.03 years. Time from onset of symptoms to disease diagnosis was 5.94 ± 3.86 years and the length of time the drug has been administered since its inception was 5.32 ± 3.74 years. Hoehn-Yahr clinical stage scores ranged from one to four, for a mean of 2.32 ± 0.81. The 12 HVs (six men) had a mean age of 40.20 ± 2.50 years. For the 40 patients (21 women) assessed in OFF and ON states (day 2), the mean age was 69.05 ± 10.54 years. Disease evolution for this subgroup was 6.38 ± 9.28 years and duration of treatment was 5.10 ± 3.52 years. The levodopa-equivalent dose was 567.09 ± 306.20 mg/day and the Hoehn-Yahr clinical stage score ranged from 1 to 3, for a mean of 2.14 ± 0.74 (Table 1).

The MDS-UPDRS-III mean score was 20.12 ± 8.60 (range 7–39), reflecting mild–moderate motor symptoms, specifically resting tremor (67.50%) and bradykinesia (35%). Speech alterations were observed in 37.50% of patients (73.33% mild and 26.67% moderate), sialorrhea and drooling were observed in 40% and 12.50% of patients, respectively, and swallowing and chewing difficulties were reported by 40% of patients (50% mild and 50% moderate). Most patients were autonomous, reporting only some slowness, difficulty, or impairment when performing routine daily activities. Cognitive status was sub-optimal and 15 patients reported occasional depression. Almost all patients had a normal nutritional status and a robust phenotype. Two thirds (66.56%) of patients reported an uncompromised quality of life at their disease stage (Table 1).

### 3.2. Pathophysiology

#### 3.2.1. Videofluoroscopy

Healthy volunteers. Swallows in all volunteers were safe (PAS 1) and effective (no oral or pharyngeal residue).

Patients with PD in ON state (*N* = 50). Impaired swallow safety (penetration) was observed in 36%, 50%, and 6% of patients for the 120 mPa·s, <50 mPa·s, and 4000 mPa·s boluses, respectively (*p* < 0.0001). Mean PAS score was 2.16 ± 1.35 and no aspirations were observed. Impaired swallow efficacy was observed in 88%, 89.58%, and 96% of patients for the 120 mPa·s, <50 mPa·s, and 4000 mPa·s boluses, respectively (ns). Piecemeal deglutition was observed in 52%, 62.50%, and 80% of patients for the 120 mPa·s, <50 mPa·s, and 4000 mPa·s boluses, respectively (*p* = 0.0207). Oral residue was recorded in 74%, 79.17%, and 82% of patients for the 120 mPa·s, <50 mPa·s, and 4000 mPa·s boluses, respectively. Vallecular residue was demonstrated in 50% of patients for the 120 mPa·s and <50 mPa·s boluses and in 61.22% of patients for the 4000 mPa·s bolus. Finally, pyriform sinus residue was found in 8%, 16.67%, and 22% of patients for the 120 mPa·s, <50 mPa·s, and 4000 mPa·s boluses, respectively.

#### 3.2.2. Oropharyngeal Physiology

Healthy volunteers. Total swallow response duration (GPJO–LVO) for 5 mL of the 120 mPa·s bolus was 753.33 ± 79.70 ms. Reconfiguration of the oropharynx from a respiratory to a digestive pathway (GPJO-LVC) lasted 156.67 ± 46.58 ms and time to opening of the UES (GPJO–UESO) was 200.00 ± 38.14 ms. Bolus propulsion by the tongue was very strong (21.50 ± 8.54 mN), leading to high bolus transit velocity (0.34 ± 0.07 m/s) and high kinematic energy (1.41 ± 0.63 mJ) (Table 2).

Patients with PD in ON state (*N* = 50). Total swallow response (GJPO-LVO) lasted 969.60 ± 216.17 ms, significantly longer than in the HVs (*p* < 0.001). Digestive-to-respiratory pathway reconfiguration was also severely delayed in comparison with the HVs, with LVC requiring 311.20 ± 102.95 ms (*p* < 0.001). In contrast, values for time to UESO (257.60 ± 200.43ms) and for propulsion strength (23.79 ± 27.98 mN), bolus velocity (0.33 ± 0.11m/s), and kinematic energy (1.62 ± 1.99 mJ) were similar to those for the HVs (Table 2).

#### 3.2.3. ROC Curves to Predict Unsafe Swallows

**Figure 3:**
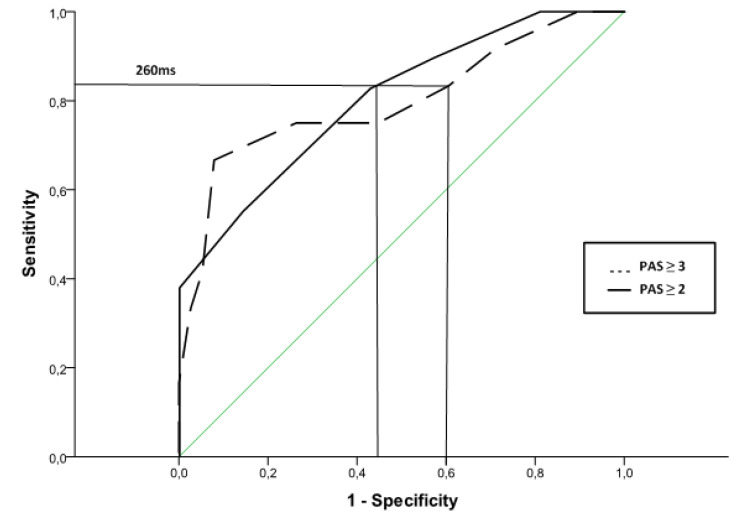
Receiver-operator characteristic (ROC) curve showing LVC time sensitivity/specificity for unsafe swallow (PAS ≥2 and PAS ≥3) of 5 mL of a 120 mPa·s bolus. Depicted is the cut-off value of 260 ms.

ROC curve analysis of LVC time was used to predict unsafe swallow for 5 mL of the 120 mPa·s (Figure 3). Considering unsafe swallow as a PAS score ≥ 3, the optimal LVC time cut-off value to predict unsafe swallow was ≥260.00 ms, for sensitivity = 0.83, specificity = 0.39, and AUC = 0.80 (95% CI: 0.68–0.92) (Figure 3). When a PAS score ≥2 was considered, the optimal LVC time cut-off value to predict unsafe swallow remained at ≥260.00 ms, for sensitivity = 0.83, specificity = 0.57, and AUC = 0.80 (95% CI: 0.63–0.97).

## 4. Impact of Dopaminergic Treatment

### 4.1. Effect of Dopaminergic Treatment on VFS Signs

Patients with PD in ON/OFF state (*N* = 40). Prevalence of safety problems was moderate, while prevalence of impaired efficacy was high, with no significant differences between the dopaminergic state of the patients (i.e., OFF vs. ON states) (Table 3).

### 4.2. Effect of Dopaminergic Treatment on Swallow Response

Patients with PD in ON/OFF state (*N* = 40)—Healthy volunteers. A comparison between the three groups was performed. Swallow response duration was similar in the OFF and ON states, and in both cases was longer than for the HVs (*p* < 0.001). Reconfiguration and time to LVC did not differ in the OFF and ON states, but both were delayed in patients with PD compared with the HVs (*p* < 0.001). There were no differences in time to UESO, bolus transit velocity, tongue propulsion strength, or kinematic energy between the OFF and ON states or in comparison with the HVs, suggesting their preservation in the early disease stage.

### 4.3. Dopaminergic Impact and the Therapeutic Effects of Thickening Agents

#### 4.3.1. Viscosity Effect: PAS and Residues

**Figure 4:**
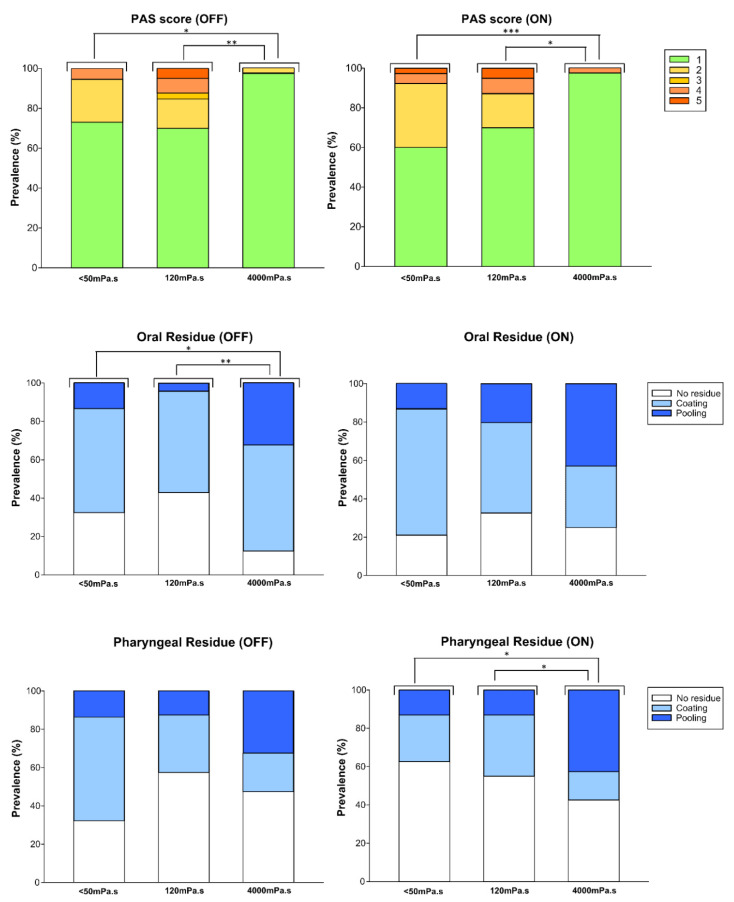
Prevalence of safe swallowing and penetration (PAS score) and oral and pharyngeal residues for three bolus viscosities (<50 mPa·s, 120 mPa·s, and 4000 mPa·s) in the OFF and ON states. OFF *p*-values * <0.05 and ** <0.01; ON *p*-values * <0.05 and *** <0.001.

**Figure 5:**
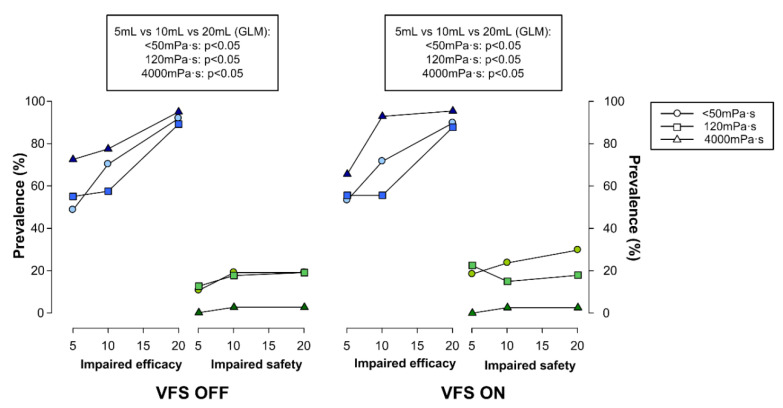
Effect of increasing bolus volume on prevalence of impaired swallow safety and efficacy for three bolus viscosities (<50 mPa·s, 120 mPa·s, and 4000 mPa·s) evaluated using videofluoroscopy (VFS).

Safety was scored for each bolus swallow using PAS scores. No penetration event in the OFF or ON states was evident for around 50% of the study population, while no aspiration was observed for any of the three viscosities (Figure 4). Increasing bolus viscosity reduced unsafe swallow prevalence in the OFF/ON groups—27.03%/39.47% (<50 mPa·s), 30%/30% (120 mPa·s), and 2.50%/2.56% (4000 mPa·s)—with significant differences between the 120 mPa·s vs. 4000 mPa·s boluses and the <50 mPa·s vs. 4000 mPa·s boluses (Figure 4). Independently of the effect of dopaminergic medication, the safest viscosity was 4000 mPa·s (Figure 5). For patients in the OFF state, there was a significant difference between the 120 mPa·s and 4000 mPa·s boluses for oral residue, with greater viscosity resulting in more oral residue. Pharyngeal residue was evident in almost half of the swallows, although no differences were encountered resulting from increases in viscosity (Figure 4). Regarding residue amount (coating and pooling), for oral residue, pooling was significantly greater for the 4000 mPa·s bolus in the OFF state (120 mPa·s vs. 4000 mPa·s; *p* = 0.031; <50 mPa·s vs. 4000 mPa·s; *p* = 0.0489). The 4000 mPa·s bolus also produced increased pooling of pharyngeal residue in the ON state (120 mPa·s vs. 4000 mPa·s: *p* = 0.0157; <50 mPa·s vs. 4000 mPa·s: *p* = 0.0288).

#### 4.3.2. Effect of Bolus Volume on Swallow (Safety and Efficacy)

We found no effect of bolus volume on swallow safety in either the ON or OFF states. In contrast, prevalence of impaired swallow efficacy in patients with PD was volume-dependent: the greater the volume, the more patients experienced impairment (Figure 5). For 120 mPa·s and for both the ON and OFF states, significant differences were found between 5 mL and 20 mL (ON *p* = 0.0010; OFF *p* = 0.0026), and between 10 mL and 20 mL (ON *p* = 0.0021; OFF *p* = 0.0026). For <50 mPa·s, there were differences for the ON state for 5 mL vs. 20 mL (*p* < 0.0001), and for 10 mL vs. 20 mL (*p* = 0.0352), while there were differences for the OFF state for 5 mL vs. 20 mL (*p* = 0.0008). Regarding 4000 mPa·s, there were significant differences for the ON state for 5 mL vs. 20 mL (*p* = 0.0129), and for 10 mL vs. 20 mL (*p* = 0.0476), and likewise, in the OFF state for 5 mL vs. 10 mL (0.0052) and 5 mL vs. 20 mL (*p* = 0.015). There was no further impact of dopaminergic treatment on the effects of bolus volume on swallow safety and efficacy.

Finally, regarding the effects of bolus volume on swallow efficacy signs in both the OFF and ON states, the GLM showed similar significant differences between the three volumes and also a significant linear trend with increased bolus volume for all three viscosities (*p* < 0.05) for both the OFF and ON states. In contrast, for both the ON and OFF states, there were no differences in swallow safety due to increasing bolus volume at any of the three viscosities (Figure 5).

## 5. Discussion

This study has described the pathophysiology and main physiological variables of OD in early PD stages, the impact of dopaminergic medication on swallowing and the effects on swallowing of three shear viscosity levels for thickened fluids.

The main finding of this study was that PD affected swallowing from early disease stages, with high and moderate prevalence of impaired efficacy and safety, respectively, and delayed oropharyngeal respiratory-to-digestive pathway reconfiguration. We found that a delay in LVC ≥260 ms was an accurate predictor of impaired safety (according to sensitivity, specificity, and AUC values). OSR was impaired in patients with early-stage PD and was not improved by dopaminergic treatment. Swallow safety was viscosity-dependent, as the higher the viscosity, the lower the prevalence of penetrations; as for swallow efficacy, this was both volume- and viscosity-dependent: the higher the volume or viscosity, the greater the prevalence and severity of residue.

We assessed the swallowing function of 50 patients with PD, 40 in the OFF and ON states. Most of the patients were elderly (older than 70 years), had been diagnosed and treated for PD for around five years, were classified as being in the mild–moderate clinical disease stage and had mild–moderate motor symptoms. Those characteristics were very similar to those of a study by Baijens [43] that, however, included only 10 patients with PD. A meta-analysis by Kalf [2] reported that the number of subjects in studies of PD varied between 44 and 1072, that most included patients were in early-intermediate disease stages, and disease duration was a minimum of five years.

Dysphagia is prevalent in PD patients with the rate depending on disease severity and diagnostic technique [2]. All patients in our study presented with impaired swallow as assessed by VFS, mainly with efficacy problems in the early swallow stages; these findings corroborate a previous study that reported 95% dysphagia prevalence and 55% impaired safety [44], while Fuh [13], in contrast, reported 63.2% prevalence for swallowing abnormalities in early PD stages. Another study [45] in which the patients with PD were placed in two groups according to the presence of dysphagia reported that patients with dysphagia (53%) were characterized by a more advanced PD stage (mean Hoehn-Yahr score 2.4 ± 0.1 vs. 1.8 ± 0.2) and longer disease duration (5.8 ± 0.7 years vs. 3.9 ± 0.5 years).

Delayed swallow response was observed in our patients, in half of whom swallow safety was mildly impaired. Baijens [43], using the same system and methodology of VFS analysis as used in our study, found normal LVC values (130 ms), thereby diverging from our results; it should be noted, however, that their study included a small number of patients.

Ours is the first study (as far as we are aware) to suggest a cut-off point related to aspiration risk in patients with PD in the early–intermediate disease stages. A delayed swallow response can be predictive of future impairment in swallow safety and thus a possible marker for early OD management in this kind of patient. Previous studies of patients with dementia [46] and stroke [47] reported similar results, except that the cut-off point for safety impairment was slightly higher (340 ms) as was the severity of the VFS signs.

In PD, impaired swallow safety increases the risk of respiratory complications and malnutrition and is also related to poor survival [48] and impaired quality of life. The use of thickening agents is a validated therapeutic strategy for patients with OD [21]. Our study throws further light on the therapeutic effect of thickened fluids for patients in early PD stages. We found that increasing bolus viscosity enhanced swallow safety independently of the dopaminergic effect; however, increasing volume impaired deglutition efficacy in our patients (as indicated by the presence of oral and pharyngeal residues), especially with the highest viscosity studied (4000 mPa·s). These results, corroborated by previous findings [8,37,49], point to the importance and the need of using suitable thickening agents and optimal doses for each OD phenotype. Depending on the composition, we can find two main groups of thickening agents: starch based and xanthan-gum based thickeners. Starch based thickeners (like that used in our study) may be more affected by salivary amylase than xanthan-gum based thickeners due to the fact that this specific enzyme breaks O-glycoside bonds, which have a higher presence in starch [41]. Another disadvantage of starch thickeners is the increase in post-deglutitive oropharyngeal residue [8,37,49] as we can observe in the results of the present study. However, not only is the composition relevant, but the optimal doses and therapeutic range offered by each thickener should also be assessed. In the present study, we tested three different viscosity levels and we could observe that 4000 mPa·s was the viscosity level needed to achieve the highest significant safety value. The problem associated with this is that we need to deal with the increment of the oral and pharyngeal residue. Our analysis showed an increase in piecemeal deglutition and pooling of oral and pharyngeal residue at the highest viscosity level assessed in our study (4000 mPa·s). In contrast, in a previous study of patients with OD and PD carried out with a mixed-composition thickener (with a high resistance to amylase), the viscosity safety threshold was reported to be maximal at 1000 mPa·s without increasing pharyngeal residue [26]. Further viscosity levels needs to be assessed to determine the therapeutic range and the optimal doses for each thickener.

The dopaminergic effect on swallowing is a rather controversial issue [16,17]. One study found no dopaminergic effects on aspiration in the early disease stages [18]; this finding agrees with our results, as we found no VFS signs that swallow response measurements were affected by dopaminergic medication. Other studies have reported partial effects on swallowing, with only 50% of patients presenting with improved swallow after medication in early disease stages [13] and in advanced disease stages [15,48]. However, the fact that studies with patients in advanced disease stages used fiberoptic endoscopic evaluation of swallowing rather than VFS may mean that their findings are not comparable to ours. Our use of VFS meant that we could also assess impaired OSR physiology [15,48]. Finally, as shown by our results, there was no dopaminergic impact on the effects of thickening agents, which, as far as we are aware, is a phenomenon that no other study has investigated.

The main limitations of this study of patients with PD are that although we included HVs, only 12 were included as controls for our 50 patients and there was no matching by age. In addition, we were only able to include 40 of our 50 patients in the OFF/ON evaluation (day 2). To our knowledge, no previous VFS studies have assessed the effects of dopaminergic medication in early–intermediate stages of PD, so we suggest that our sample size is adequate to draw meaningful conclusions. Another limitation was not being able to randomize the order to perform the VFS in the OFF or ON state because the study design was adapted to minimize the possible patients’ discomfort during the 12 h period needed to achieve the OFF status and to come more than twice to the hospital.

## 6. Conclusions

OSR is severely delayed in early disease stages in patients with PD compared to HVs. Mildly impaired swallow safety improved with increased bolus viscosity, while swallow efficacy (as reflected in oral and pharyngeal residues) was increasingly impaired with increasing volumes and viscosities (based on starch-based thickeners). Neither swallowing function nor the therapeutic effect of thickening agents were impacted by dopaminergic treatment. Although this information provides evidence in relation to the controversy surrounding the effect of this treatment for patients with PD and OD, more studies with new generation xanthan gum thickeners are necessary to fully assess the effect of dopaminergic drugs in more advanced PD stages.

## Figures and Tables

**Table 1:** Demographic data, comorbidities, and clinical status of patients with Parkinson’s disease.

		N (%) /Mean ± SD (Range)
Gender (*N* = 50)	Men	26 (52.00)
	Women	24 (48.00)
Comorbidities (*N* = 40)	Arthrosis/rheumatism	21 (52.50)
	Depression	7 (17.50)
	Hypertension	17 (42.50)
	Dyslipidemia	14 (35.00)
Hoehn-Yahr clinical stage (*N* = 50)	1	8 (16.00)
	1.5	2 (4.00)
	2	14 (28.00)
	2.5	8 (16.00)
	3	15 (30.00)
	4	3 (6.00)
MNA-SF (*N* = 40)	At risk of malnutrition	4 (10.00)
	Normal nutrition	36 (90.00)
Frailty criteria (*N* = 40)	Robust	33 (82.50)
	Pre-frail	7 (17.50)
ADL (*N* = 40)		87.25 ± 11.09 (60–100)
PDQ-8 (*N* = 40)		4.8 ± 5.01 (0–19)
MDS-UPDRS-III (*N* = 40)		20.12 ± 8.60 (7–39)
MoCA (*N* = 40)		24.12 ± 4.32 (13–30)

ADL, Schwab-England Activities of Daily Living; MDS-UPDRS-III, Movement Disorder Society-Sponsored Revision of the Unified Parkinson Disease Rating Scale (Part III); MNA-SF, Mini Nutritional Assessment Short Form; MoCA, Montreal Cognitive Assessment; PDQ-8, Parkinson Disease Questionnaire-8.

**Table 2:** Oropharyngeal swallow response as evaluated by videofluoroscopy in patients with Parkinson’s disease (PD) in the ON state and healthy volunteers (HVs).

Oropharyngeal Swallow Response (Mean ± SD)	HVs	PD (*N* = 50)	*p*-Value
LVC (ms)	156.67 ± 46.58	311.20 ± 102.95	<0.0001
UESO (ms)	200.00 ± 38.14	257.60 ± 200.43	0.2009
LVO (ms)	753.33 ± 79.70	969.60 ± 216.17	<0.0001
Kinematics (mJ)	0.34 ± 0.07	0.33 ± 0.17	0.2811
Force (mJ)	1.41 ± 0.63	1.62 ± 1.99	0.2444
Mean bolus velocity (m/s)	21.50 ± 8.54	23.79 ± 27.98	0.3141

LVC, laryngeal vestibule closure; LVO, laryngeal vestibule opening; PAS, Penetration–Aspiration Score; UESO, upper esophageal sphincter opening.

**Table 3:** Videofluoroscopy (VFS) signs of impaired swallow safety and efficacy and oropharyngeal swallow response for patients with Parkinson’s disease (PD) in dopaminergic OFF and ON states.

	Patients with PD		
	OFF (*N* = 40)	ON (*N* = 40)	*p*-Value
Impaired swallow efficacy (%)	97.50 (39)	100.00 (40)	0.4937
Liquid (<50 mPa·s)	91.89 (34)	86.84 (33)	1.000
Nectar (120 mPa·s)	85.00 (34)	85.00 (34)	1.000
Pudding (4000 mPa·s)	92.50 (37)	94.87 (37)	1.000
**Oral residue**			
Coating	60.00 (24)	42.50 (17)	0.6830
Pooling	32.50 (13)	47.50 (19)	1.0000
**Pharyngeal residue**			
Coating	20.00 (8)	20.00 (8)	1.0000
Pooling	40.00 (16)	45.00 (18)	0.7205
Impaired swallow safety (%)	37.50 (15)	47.50 (19)	0.4978
Liquid (<50 mPa·s)	27.03 (10)	39.47 (15)	0.3289
Nectar (120 mPa·s)	30.00 (12)	30.00 (12)	1.000
Pudding (4000 mPa·s)	2.50 (1)	2.50 (1)	1.000
Penetrations (%)	37.50 (15)	47.50 (19)	0.4978
Aspirations (%)	0	0	-
Silent aspirations (PAS = 8) (%)	0	0	-
Higher mean PAS	1.73 ± 1.20	1.90 ± 1.28	>0.05
**Oropharyngeal swallow response (mean ± SD)**			
LVC (ms)	293.33 ± 90.07	305.00 ± 105.90	>0.05
UESO (ms)	211.28 ± 55.02	219.00 ± 77.92	>0.05
LVO (ms)	953.85 ± 166.59	938.00 ± 122.52	>0.05
Kinematics (mJ)	0.34 ± 0.11	0.35 ± 0.14	>0.05
Force (mJ)	1.54 ± 1.17	1.65 ± 1.53	>0.05
Mean bolus velocity (m/s)	22.45 ± 15.15	24.53 ± 23.57	>0.05

LVC, laryngeal vestibule closure; LVO, laryngeal vestibule opening; PAS, Penetration-Aspiration Score; UESO, upper esophageal sphincter opening.
